# Immunoproteomics approach revealed elevated autoantibody levels against ANXA1 in early stage gallbladder carcinoma

**DOI:** 10.1186/s12885-020-07676-6

**Published:** 2020-12-01

**Authors:** Javed Akhtar, Ratna Priya, Vaishali Jain, Puja Sakhuja, Anil Kumar Agarwal, Surbhi Goyal, Ravindra Varma Polisetty, Ravi Sirdeshmukh, Sudeshna Kar, Poonam Gautam

**Affiliations:** 1 Laboratory of Molecular Oncology, ICMR- National Institute of Pathology, Safdarjung Hospital Campus, New Delhi, 110029 India; 2 Jamia Hamdard- Institute of Molecular Medicine, Jamia Hamdard, New Delhi, 110062 India; 3 Manipal Academy of Higher Education (MAHE) , 576104 Manipal, India; 4 Govind Ballabh Pant Institute of Postgraduate Medical Education and Research (GIPMER), New Delhi, 110002 India; 5 Department of Biochemistry, Sri Venkateswara College, University of Delhi, New Delhi, 110021 India; 6 Institute of Bioinformatics, International Tech Park, Bangalore, 560066 India

**Keywords:** Autoantibody, Gallbladder carcinoma, Immunoproteomics, ANXA1, HSPD1

## Abstract

**Background:**

Early diagnosis is important for the timely treatment of gallbladder carcinoma (GBC) patients and may lead to increased survival outcomes. Here, we have applied serological proteome analysis (SERPA), an immunoproteomics approach, for the detection of ‘tumor-associated antigens (TAAs) that elicit humoral response’ in early stage GBC patients.

**Methods:**

Total protein from pooled tumor tissue of GBC patients (*n* = 7) was resolved by two-dimensional gel electrophoresis (2-DE) followed by immunoblotting using pooled blood plasma from healthy volunteers (*n* = 11) or gallstone disease (GSD) cases (*n* = 11) or early stage GBC (Stage I and II) (*n* = 5) or GBC stage IIIA (*n* = 9). 2-D gel and immunoblot images were acquired and analyzed using PDQuest software to identify immunoreactive spots in GBC cases in comparison to controls. Proteins from immunoreactive spots were identified by liquid chromatography- tandem mass spectrometric analysis (LC-MS/MS). Autoantibody levels for two of the functionally relevant proteins were investigated in individual plasma samples (52 cases and 89 controls) by dot blot assay using recombinant proteins.

**Results:**

Image analysis using PDQuest software identified 25 protein spots with significantly high or specific immunoreactivity in GBC cases. Mass spectrometric analysis of 8 corresponding protein spots showing intense immunoreactivity (based on densitometric analysis) in early stage GBC or GBC stage IIIA cases led to the identification of 27 proteins. Some of the identified proteins include ANXA1, HSPD1, CA1, CA2, ALDOA and CTSD. Among the two proteins, namely ANXA1 and HSPD1 verified using a cohort of samples, significantly elevated autoantibody levels against ANXA1 were observed in early stage GBC cases in comparison to healthy volunteers or GSD cases (unpaired t-test, *p* < 0.05). Receiver operating characteristic (ROC) curve analysis for ANXA1 showed an Area under the Curve (AUC) of 0.69, with 41.7% sensitivity against a specificity of 89.9% for early stage GBC. IHC analysis for ANXA1 protein showed ‘high’ expression levels in 72% of GBC cases whereas all the controls showed ‘low’ expression levels.

**Conclusions:**

The study suggests that the ANXA1 autoantibody levels against ANXA1 may be potentially employed for early stage detection of GBC patients. Other proteins could also be explored and verified in a large cohort of clinical samples.

**Supplementary Information:**

The online version contains supplementary material available at 10.1186/s12885-020-07676-6.

## Background

Gallbladder carcinoma (GBC) is the fifth most common and aggressive malignancy of the gastrointestinal tract, with a high prevalence and incidence rate in Latin America (Chile) and Asian region (northern and northeast India) [1, 2]. Gallstone disease (GSD) is one of the major risk factors for GBC [3]. GBC is generally presented at the advanced stages due to its anatomic position and non-specificity of symptoms at early stages. Complete surgical resection is the potentially curative treatment for GBC at initial presentation with better survival outcomes, however, patients with metastatic GBC have a poor prognosis [4]. Five-year survival rate for early stages is higher (70–90% in Stage I and 45–60% in Stage II, when treated with extended cholecystectomy) in comparison to advanced stage GBC patients (≤20%) [5]**.** Therefore, the detection of GBC at an early stage may significantly improve the survival of these patients.

Antibodies against tumor-associated antigens (TAAs) have been reported in the serum of patients with various types of cancer and showed potential as biomarkers for early detection of cancer [6]. TAAs are generally present in low amounts at early stages and may not be detected by the available technologies; however, the autoantibodies generated against these TAAs are present in a high amount and may be detected in a pre-malignant stage. During the early stages of tumor development, these TAAs (proteins or peptides) may originate due to alterations at the genetic or protein levels and elicit humoral response [7]. As autoantibodies are highly stable in serum and are not proteolyzed, the detection of anti-TAA autoantibodies has the potential to improve assays for early detection of cancer [8].

Serological proteome analysis (SERPA), an immunoproteomics approach based on a classical proteomics workflow, is one of the most common tools that involve two-dimensional gel electrophoresis (2-DE), western blot and mass spectrometry analysis. The main advantage of using this method is the simultaneous analysis of whole protein, post-translational modifications and protein isoforms [9]. SERPA approach has been used for the identification of TAAs in several cancers including gingivo buccal complex cancer [10], hepatocellular carcinoma (HCC) [11], breast cancer [12]. Mustafa et al identified 18 and 9 immunoreactive spots, detected in atleast 4 out of 13 cholangiocarcinomas (CCA) sera, from the lysate of two CC cell lines and tumor tissue lysate respectively using this approach. They further validated anti-vimentin and anti-actin antibodies on colchicine-treated Hep2 cells and confirmed increased levels in 8 (61%) and 3 (23%) out of 13 CC cases respectively [13]**.** However, to the best of our knowledge, there is no report available on the identification of TAAs eliciting humoral response in GBC, till date.

The present study applies SERPA approach for detection of TAAs eliciting humoral response in GBC cases. Verification of two of the identified TAAs in a large cohort of clinical samples using Dot blot assay reveals ANXA1 as a candidate TAA to be considered further for clinical applications.

## Methods

### Clinical samples

Adult patients (with age ≥ 20 years) diagnosed with GBC or GSD cases visiting Govind Ballabh Pant Institute of Postgraduate Medical Education and Research (GIPMER), New Delhi, were recruited for the study after approval from the Institutional Human Ethics Committee. Tumour Staging was done on the basis of clinical data of patients, histopathological evaluation and imaging tools, as per AJCC, 8th edition staging system [14]. Tissue samples from GBC cases (*n* = 7) and blood plasma samples from GBC cases (*n* = 52), GSD cases with no dysplasia (*n* = 48) and healthy volunteers (*n* = 41) were used in this study. Clinico-pathological data of these subjects are detailed in Table 1. Clinical parameters for the patients, wherever available (~ 70%), such as white cell count, liver enzymes (SGOT/SGPT/ALP) and cholestasis were collected.

**Table 1:** Clinico-pathological data of case and control subjects. (A) Tissue samples and (B) Plasma samples used for the study

Subjects	Total number	Number of males	Number of females	Mean age (Years)	Age range (years)
**(A) Tissue samples**					
***Total GBC Cases***	**7**	**0**	**7**	**50.86**	**30–66**
Stages					
GBC, Stage I	1	0	1	38	38
GBC, Stage II	1	0	1	65	65
GBC, Stage IIIA	5	0	5	50.6	30–66
Histological grade					
Well-differentiated (G1)	1	0	1	–	–
Moderately-differentiated (G2)	4	0	4	–	–
Poorly-differentiated (G3)	2	0	2	–	–
**(B) Plasma samples**					
***Total GBC Cases***	**52**	**10**	**42**	**50.88**	**22–78**
Stages					
GBC, Stage I	6	1	5	39.66	22–47
GBC, Stage II	6	1	5	52.16	34–66
GBC, Stage III	17	2	15	52.76	30–66
GBC, Stage IV	23	6	17	52.08	38–78
Early Stages (I and II)	12	2	10	45.91	22–66
Advanced stages (III and IV)	40	8	32	52.37	30–78
LN status					
LN negative	23	2	21	48.91	22–66
LN positive	29	8	21	52.44	38–78
Histological grade					
Well-differentiated (G1)	5	0	5	–	–
Moderately-differentiated (G2)	32	5	27	–	–
Poorly-differentiated (G3)	15	2	13	–	–
***Total Controls***	**89**	**28**	**61**	**40.89**	**20–72**
GSD cases	48	13	35	43.06	20–72
Healthy group	41	15	26	38.36	24–59

*GBC* Gallbladder carcinomas, *GSD* Gallstone disease, *LN* Lymph node

Tissue samples were collected immediately after surgical resection from patients with GBC and stored at − 80 °C until used for further analysis. Peripheral blood (Approx. 5 ml) was collected in EDTA vials from patients with pre-operative GBC, pre-operative GSD and healthy volunteers. These blood samples were centrifuged at 2500×g for 15 min at 4 °C, clear plasma separated, aliquoted and stored at − 80 °C. All the samples were processed within 30 min of collection.

For in-house tissue microarrays (TMAs) preparation, formalin-fixed paraffin-embedded (FFPE) tissue samples from GBC cases and controls [Healthy individuals (gallbladder tissue from liver donors) and GSD cases] were drawn from GIPMER, New Delhi, India and National Liver Disease Biobank- Institute of Liver and Biliary Sciences (NLDB-ILBS), New Delhi, India, after approval from the Institutional Human Ethics Committee. Liver donors are donating healthy right lobe of the liver and the gallbladder is removed at the time of surgery. The details of the TMA preparation and samples used are described under ‘Immunohistochemistry analysis’ section.

### Protein extraction from GBC tissue samples

Pooled tumor tissue (50 mg from seven GBC patients with Stage I, II and IIIA) was grinded in liquid nitrogen and added RIPA buffer [25 mM Tris-Cl, pH 7.6 + 150 mM NaCl + 2% (3-[(3-cholamidopropyl) dimethylammonio]-1-propanesulfonate (CHAPS)] with 1% protease inhibitor cocktail (Sigma, USA). The tissue homogenate was then sonicated (three bursts of 10 s each with 10 s of pause interval at 4 °C) and centrifuged at 13,500×g for 20 min at 4 °C. The supernatant was separated and protein was quantified by Bradford assay. Sodium dodecyl sulphate- polyacrylamide gel electrophoresis (SDS-PAGE) was performed to analyze the protein profile of tissue lysate.

### Immunodepletion of GBC tissue lysate

Tissue lysate was subjected to immunodepletion using Multiple Affinity Removal Spin Cartridge, human serum albumin (HSA)/immunoglobulin (Ig) G (Agilent Technologies, USA) to remove two of the abundant proteins (Albumin and IgG), as per the instructions by the manufacturer. Protein quantification followed by SDS-PAGE analysis was performed to confirm the removal of abundant proteins.

### 1-D immunoblot analysis

For 1-D immunoblot analysis, immunodepleted GBC tissue proteins were separated by SDS-PAGE and electrotransferred to polyvinyl difluoride (PVDF) membrane. The blots were blocked with 5% skimmed milk powder in TBST [1× tris buffered saline (10 mM Tris-Cl, pH 7.4 and 30 mM NaCl) with 0.05% Tween 20 and 0.005% Triton-X-100] at RT for 1 h. The blots were then incubated with pooled blood plasma from healthy individuals (*n* = 11) or GSD cases (*n* = 11) or GBC Stage I and II (*n* = 3) or GBC Stage IIIA (*n* = 9) (1:1000 dilution) at 4 °C overnight. The blots were then incubated with anti-human IgG conjugated with horseradish peroxidase (HRP) (1:40,000 dilution) (Thermo Scientific, USA) at RT for 1 h and were developed using the enhanced chemiluminescent (ECL) Kit (Millipore, USA). The images were acquired using Chemidoc MP imager and immunoblots were analyzed using Image Lab 4.1 software (Bio-Rad).

### 2-D immunoblot analysis

The immunodepleted GBC tissue proteins were separated using 2-DE method as described by Gorg et al [15]. Briefly, a total of 120 μg protein which is dissolved in rehydration buffer [7 M Urea+ 2 M Thiourea + 4% CHAPS + 1% dithiothreitol (DTT) + 1% N-Decanoyl-N-methylglucamine (MEGA 10) with 0.2% bio-lyte with pI range 3–10 and 0.002% bromophenol blue] was used for passive rehydration of immobilized-pH-gradient (IPG) strip, 11 cm, 3–10 NL (Bio-Rad, USA). Proteins were separated in the first dimension on Protean i12 isoelectric focusing (IEF) system (Bio-Rad, USA) at 20 °C using the following conditions: 250 V for 20 min; 8000 V (gradual mode) for 1 h; 8000 V (rapid mode) for an additional 26,000 Vh; 750 V (rapid) on hold with a maximum current of 50 μA/IPG strip. After IEF, the IPG strips were incubated in equilibration buffer [6 M urea, 30% glycerol, 2% sodium dodecyl sulphate (SDS), 50 mM Tris-HCl buffer, pH 8.8)] with 2% DTT for 15 min and then in equilibration buffer with 5% iodoacetamide for 15 min (Bio-Rad protocol). The proteins were further resolved in the second dimension by SDS-PAGE (4–20% gradient midi gel) (Bio-Rad, USA) initially at 50 V for 30 min and then at 100 V until the dye front reached the bottom of the gel. The gel was stained with Coomassie Brilliant Blue R250 to visualize the proteins.

For 2-D immunoblot analysis, immunodepleted GBC tissue proteins (120 μg) were separated in the first dimension by IEF (IPG strip 11 cm, 3–10 NL) and then in the second dimension by SDS-PAGE (4–20% gradient gel) as described above. The proteins were then electrotransferred to the PVDF membrane using a semi-dry method (Bio-Rad). The blots were blocked with skimmed milk powder followed by incubation with pooled blood plasma from healthy individuals (*n* = 11) or GSD cases (*n* = 11) or GBC Stage I and II (*n* = 5) or GBC Stage IIIA (*n* = 9) (1:1000 dilution). The blots were then incubated with anti-human IgG conjugated with HRP (Thermo) (1:40,000 dilution) and were developed using ECL Kit (Millipore). All other conditions were the same as that of 1-D immunoblot analysis. The images were acquired using the ChemiDoc MP imaging system (Bio-Rad) and immunoblots were analyzed to identify protein spots showing immunoreactivity in GBC cases.

### In-gel digestion

The 2-D immunoblot images were compared with 2-D gel images (Coomassie Stained) to identify corresponding immunoreactive protein spots on the 2-D gel using PDQuest software version 8.1. The coomassie-stained protein spots of interest were excised using glass capillaries (internal diameter ~ 1 mm) and incubated with 400 μl of destaining solution [25 mM NH_4_HCO_3_ in 50% acetonitrile (ACN)] in microfuge tube at RT. The destaining solution was changed after every 15 min till the gel pieces were completely destained followed by the addition of 400 μl of 100% ACN and incubated for 5 min. ACN was removed and dried the gel using a speed vacuum concentrator followed by the addition of 15 μl of 10 ng/μl trypsin (prepared in 25 mM NH_4_HCO_3_) to the dehydrated gel and incubated overnight at 37 °C. The next day, 50 μl of peptide extraction buffer [0.3% Trifluoroacetic acid (TFA) in 50% ACN] was added to each tube and incubated for 30 min with gentle shaking. The solution was then removed and stored in a fresh microfuge tube. This step was repeated twice and the solution was pooled with the previous tube and lyophilized to get trypsin digested peptides.

### Mass spectrometric analysis

Tryptic digests from different protein spots were subjected to mass spectrometric analysis (nanoRPLC-MS/MS) for protein identification. Nanoflow electrospray ionization tandem mass spectrometric analyses of peptide samples were carried out using Orbitrap QExactive plus (Thermo Scientific, Bremen, Germany) coupled with RS nano high-performance liquid chromatography (HPLC) 3000 system (Thermo Scientific, Bremen, Germany). Peptide samples were enriched using a C18 Trap column and separated on an Acclaim™ PepMap™ 100 C18 analytical column (3 μm, 150 mm, 0.075 mm I.D.) at a flow rate of 300 nl/min using a linear gradient of 7–30% ACN over 65 min. The Mass spectrometric analysis was carried out in a data-dependent manner with full scans acquired using the Orbitrap mass analyzer at a mass resolution of 70,000 at m/z 200. From each MS scan, 20 most intense precursor ions were selected for MS/MS fragmentation and detected at a mass resolution of 35,000 at m/z 200. The fragmentation was carried out using higher-energy collision dissociation (HCD) with 30% normalized collision energy. The ions selected for fragmentation were excluded for 30 s. The automatic gain control for full FT-MS was set to 1 million ions and FT-MS/MS was set to 0.1 million ions with a maximum time of accumulation of 500 ms. For accurate mass measurements, the lock mass option was enabled.

### Data analysis

Protein identifications were carried out as follows. The MS data were analyzed using Proteome Discoverer (Thermo Fisher Scientific, Version 1.4). MS/MS search was carried out using the SEQUEST search engine against the NCBI RefSeq database version 81. Search parameters included trypsin as an enzyme with 1 missed cleavage allowed; precursor and fragment mass tolerance were set to 20 ppm and 0.1 Da, respectively; Methionine oxidation was set as a dynamic modification while S-carboamidomethyl modification at cysteine was set as static modifications. The false discovery rate (FDR) was calculated by enabling the peptide sequence analysis using a decoy database. High confidence peptide identifications were obtained by setting a target FDR threshold of 1% at the peptide. For the identification of peptides/ proteins, signal to noise ratio applied was 1.5 or more and this is within the acceptable standards for the instrumentation platform used. Contaminant peaks such as peaks of keratin proteins were selected under the exclusion list. Proteins identified by ≥2 unique peptides were considered as high confidence identifications. Molecular functions and localization of the identified proteins were derived from the human protein reference database, http://www.hprd.org, [16] and UniProt database, www.uniprot.org/uniprot.

### Dot blot assay

Dot blot assay was performed for Annexin A1 (ANXA1) and heat shock protein 60 (HSPD1) to analyze the autoantibody levels in individual plasma samples. A total of 75 ng recombinant human ANXA1 protein or HSPD1 (Abcam, USA) was spotted on the PVDF membrane. The blots were blocked with 5% skimmed milk powder in 1× TBST followed by incubation with individual blood plasma from healthy individuals (*n* = 41), GSD cases (*n* = 48), GBC Stage I and II (*n* = 12), GBC Stage IIIA (*n* = 11), GBC Stage IIIB (*n* = 6) and GBC Stage IVB (*n* = 23) (dilution 1:1000). The blots were then incubated with secondary antibody (anti-human IgG HRP conjugated) (1:40,000 dilution) followed by development using SuperSignal™ West Femto Maximum Sensitivity Substrate (Thermo, USA). All other conditions were the same as that of 1-D immunoblot analysis. The images were acquired using Chemidoc MP (Bio-Rad, USA) and densitometric analysis was performed using Image Lab software version 4.1 (Bio-Rad, USA) to compare the autoantibody levels in controls and cases. Two of the plasma samples were included in all the dot blot experiments and were used for normalization.

### Statistical analysis

Statistical analysis was performed using GraphPad Prism 5. The autoantibody levels, indicated by density (arbitrary units) of immunoreactive spots of individual controls and cases were used for the analysis. Differences in autoantibody levels between two independent groups were tested with unpaired t-test (two-tailed) with confidence intervals of 95% and *p*-value of less than 0.05 was used to indicate statistical significance. The receiver operating characteristic (ROC) analysis for autoantibody levels against ANXA1 for various groups of GBC [Early stage (GBC stage I and II) vs healthy or GSD; Stage IIIA vs healthy or GSD; stage IIIB vs healthy or GSD; stage IVB vs healthy or GSD; early stage vs all controls; advanced stage (stage III and IV) vs all controls; LN negative GBC vs all controls; LN positive vs all controls] was performed leading to the estimates of the area under the curve (AUC) with 95% confidence interval (CI) along with sensitivity and specificity. The autoantibody levels below the cut-off value were considered ‘low’ and above the cut-off value was considered as ‘high’.

### Immunohistochemistry analysis

IHC was performed on FFPE tissues using tissue microarray (TMAs) and individual tissue sections to analyze the expression of ANXA1 protein. In-house TMAs were prepared as follows. Two TMA blocks were constructed using the FFPE blocks and included 14 controls (2 healthy liver donors and 12 GSD cases) and 31 GBC cases (9 early stage and 22 advanced stage). Each TMA block consisted of 22 cores of 2 mm diameter, and Hematoxylin and Eosin (H & E) - stained sections of the blocks were used to define tumor regions. Further, 4 μm sections were cut from the TMA block for carrying out IHC. Individual tissue sections (FFPE) of GBC (7 early stage and 5 advanced stage) were also used for IHC analysis. In brief, after deparaffinization and rehydration of FFPE tissue sections, antigen retrieval was performed by immersing the slide in antigen retrieval buffer (20 mM Tris buffer, pH 9.0) at 90 °C for 20 min. Endogenous peroxidases were blocked with 0.03% hydrogen peroxide, and nonspecific binding was blocked with protein blocking reagent. Sections were then incubated for 1 h at RT with primary antibody against ANXA1 (dilution 1:4000, Cat. No. ab214486, Abcam, USA) followed by incubation with PolyExcel PolyHRP for 40 min at RT. Tissue sections were then incubated with Stunn DAB working solution for 5 min at RT (PathnSitu Biotechnologies, USA). Sections were counter stained with Mayer’s hematoxylin, dehydrated and images were taken under the microscope. The distribution of staining and staining intensity across the section was observed under the microscope. Scoring criteria were based on both staining intensities and distributions. The staining intensity of cancer cells scored as 0, 1+, 2+/3+ indicating negative, low, and strong staining respectively. The distribution of staining of cancer cells was scored as 0 (< 10% of cells staining), 1+ (10- < 25% of cell staining), 2+ (25- < 50% of cells staining) and 3+ (≥50% of cells staining). ANXA1 expression was considered ‘high’ if the percentage distribution was ≥25% and ‘low’ if it was < 25%. IHC data analysis was analyzed by two independent pathologists.

## Results

**Fig. 1:**
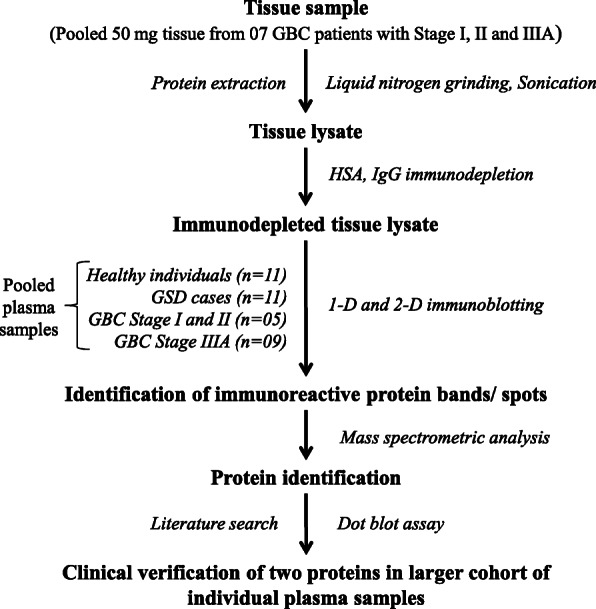
Workflow to study autoimmune response in GBC patients. GBC- Gallbladder carcinoma; HSA- Human serum albumin

In the present study, we have used immunoproteomics approach for the detection of TAAs, eliciting a humoral response in GBC patients, followed by clinical verification by dot blot assay using recombinant proteins. The overall workflow of the study is shown in Fig. 1.

### Clinical parameters

The GBC samples (*n* = 52) used for the study included 23% early stage GBC cases (*n* = 12) and ~ 80% female patients (*n* = 42) as shown in the demographic data**.** A total of 23 cases were lymph node (LN) negative and 29 were LN positive (Table 1a). Among the GBC cases used for pooling of tissue samples, we find variations among the samples irrespective of their stage. There was no significant variations among the GSD and GBC early stage (stage I and II) used for pooling of plasma, however, there were variations among the GBC stage IIIA cases (Supplementary Figure S1). Analysis of the clinical parameters in GSD and GBC cases used for verifications (individual plasma) showed variations in clinical parameters among both the groups (Supplementary Figure S1).

### Autoantibody response analysis in GBC

**Fig. 2:**
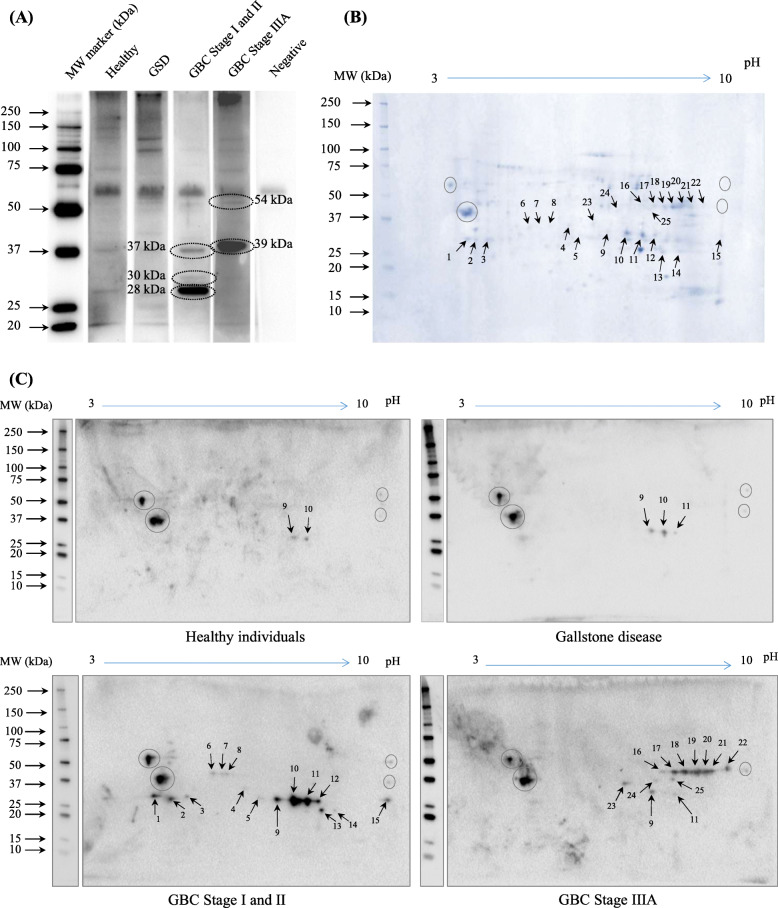
Autoantibody response analysis in GBC using 1-D and 2-D immunoblotting. Pooled immunodepleted GBC tissue lysate was resolved by SDS–PAGE and electro-transferred to PVDF membrane followed by blocking and incubation with pooled plasma from healthy individuals (*n* = 11), gallstone cases (*n* = 11), GBC Stage I and II (*n* = 5), and GBC Stage IIIA (*n* = 9) (dilution 1:1000). The blots were then incubated with secondary antibody (anti-human IgG HRP conjugated, dilution 1:40,000) followed by development using ECL reagent. All images were acquired using Chemidoc MP (Bio-Rad). (**a)** Image analysis of 1-D immunoblots showed 03 protein bands (37 kDa, 30 kDa and 28 kDa) with high or specific immunoreactivity in GBC Stage I and II, and 02 protein bands (54 kDa and 39 kDa) with high or specific immunoreactivity in GBC Stage IIIA (encircled). (**b)** Coomassie-stained 2-D gel images and (**c**) 2-D immunoblot images were analyzed by PDQuest software (Bio-Rad) which showed 25 protein spots (marked with an arrow) with specific reactivity in GBC cases. The proteins showing non-specific reactivity both in cases and controls are marked by circle. The full-length blot images are presented in Supplementary Figure S3

The 1-D Immunoblot analysis with immunodepleted GBC tissue lysate probed with pooled plasma from healthy individuals or GSD or early stage GBC (stage I and II) or GBC Stage IIIA showed 03 protein bands (37 kDa, 30 kDa and 28 kDa) with high or specific immunoreactivity in early stage GBC, and 02 protein bands (54 kDa and 39 kDa) with high or specific immunoreactivity in GBC Stage IIIA (Fig. 2a, Supplementary Figure S3A). Further, 2-D immunoblot analysis using the same pool of plasma samples as above showed significantly high or specific reactivity for a total of 25 protein spots in GBC cases. These spots are marked with an arrow in the Coomassie-stained 2-D gel and 2-D immunoblots (see Fig. 2b and c, Supplementary Figure S3B). A total of 13 protein spots were found particularly in early stage and 10 protein spots in Stage IIIA, and 2 were common (spot no. 9 and 11) among both the groups.

### Protein identification

Liquid chromatography-tandem mass spectrometric analysis (LC-MS/MS) of the four most intense immunoreactive protein spots (based on the densitometric analysis) in early stage GBC (Spot No. 9–12) or in GBC Stage IIIA (Spot No. 17–20), led to the identification of 17 and 12 proteins respectively (Fig. 2, Supplementary Table S1). The list of identified proteins and corresponding peptides is shown in Supplementary Table S2. We performed a literature search to assess the significance of the individual proteins for further clinical verifications. The ‘molecular functions’ of these proteins mainly include catalytic activity and transporter activity whereas the ‘biological processes’ include energy metabolism and transport. These proteins were majorly localized to the cytoplasmic, plasma membrane, extracellular (Supplementary Table S3). Some of the important proteins identified from immunoreactive spots in early stage GBC include carbonic anhydrase isoform 1 and 2 (CA1 and CA2), ANXA1, HSPD1, aldolase A and B (ALDOA and ALDOB), cathepsin D preproprotein (CTSD) and plectin isoform 1d (PLEC) and those identified from immunoreactive spots in GBC Stage IIIA include ALDOA, ALDOB, arginase 1 (ARG1) and lactotransferrin isoform 2 (LTF). Two of the proteins, ALDOA and ALDOB were common among both the groups. Identification of multiple proteins from the individual gel spots could be presumably due to lower resolution of proteins through broad pH range used for iso-electric focusing in 2-DE.

Similarly, we also observed some individual proteins detected in multiple spots. For examples, among early stage GBC, five proteins [ALDOB, CA1, hemoglobin subunit beta (HBB), immunoglobulin lambda-like polypeptide 5 isoform 1 (IGLL5), PLEC] were identified in ≥3 protein spots, while five proteins [ANXA1, CA2, CTSD, delta (3,5)-Delta (2,4)-dienoyl-CoA isomerase, mitochondrial precursor (ECH1), hydroxyacylglutathione hydrolase, mitochondrial isoform 2 (HAGH)] were identified in ≥2 protein spots.

Among GBC stage IIIA, three proteins (ALDOA, ALDOB and LTF) were identified in ≥3 protein spots while one protein, catalase (CAT), was identified in ≥2 protein spots (Supplementary Table S3). Detection of the same protein in multiple spots could be due to the existence of their isoforms, although we do not have any evidence for this.

### Clinical verification by dot blot assay

**Fig. 3:**
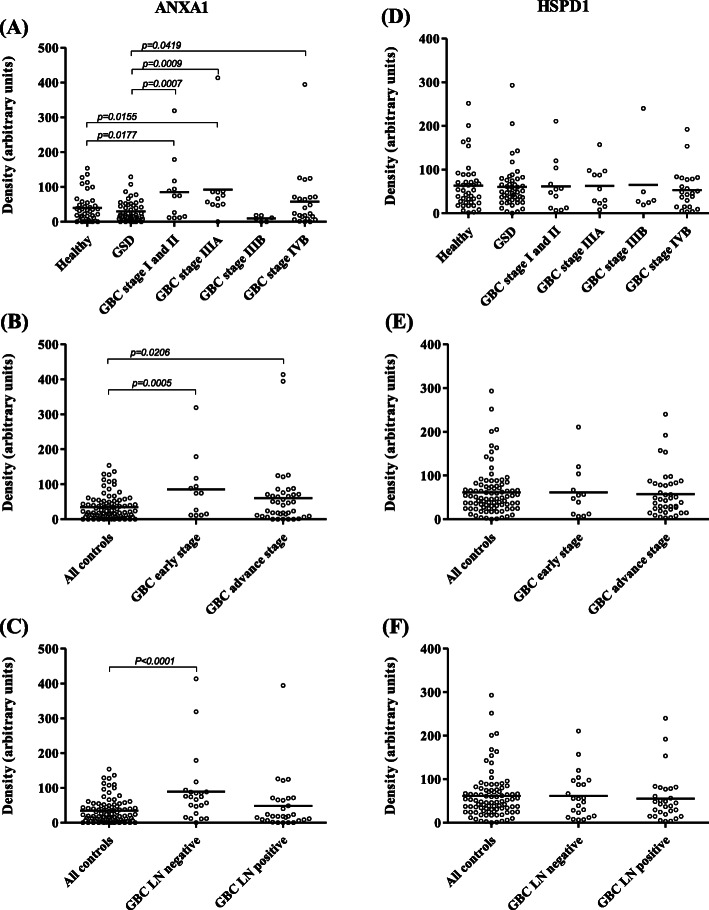
Clinical verification of ANXA1 and HSPD1 by Dot blot assay. Scatter plot showing relative levels of autoantibodies in controls (Healthy individuals, *n* = 34; GSD, *n* = 48) and cases (GBC stage I and II, *n* = 12; GBC Stage IIIA (*n* = 11); GBC Stage IIIB (*n* = 6); GBC stage IVB, *n* = 23). (**a)** The analysis showed a significant difference (*p* value ≤0.05) in autoantibody levels for ANXA1 in GBC stage I and II or IIIA (**b**) early stage GBC cases and (**c**) lymph node negative GBC cases (**c**) in comparison to both the controls. No significant difference in autoantibody levels for HSPD1 was observed in any of the GBC stages (**d**), early or advanced stage GBC cases (**e**) and lymph node negative or positive cases (**f**) in comparison to controls

**Fig. 4:**
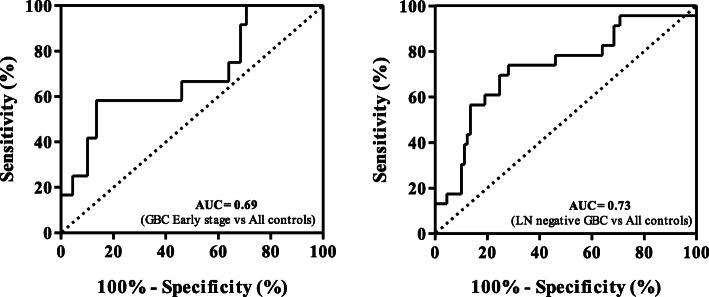
Receiver operating characteristic (ROC) curve for ANXA1 antibodies in plasma from GBC cases and controls. (**a)** ROC curve analysis showed an Area under the ROC Curve (AUC) of 0.69 (95% CI: 0.52–0.86) with 41.7% sensitivity against a specificity of 89.9% in early stage GBC in comparison to controls and (**b)** an AUC of 0.73 (95% CI: 0.61–0.85) with 30.4% sensitivity against a specificity of 89.9% in lymph node negative GBC in comparison to controls

Due to the presence of multiple proteins in a given gel spot, clinical verification was performed for two of the important proteins, ANXA1 and HSPD1, identified from protein spots showing immunoreactivity in early stage GBC cases (Supplementary Figures S2 and S3C). Clinical verification using individual blood plasma from 52 GBC cases (GBC stage I, II, III and IV) and 89 controls (healthy volunteers and GSD cases) (Table 1) showed significantly high autoantibody levels for ANXA1 in early stage GBC (stage I and II) as well as in GBC Stage IIIA (*p* value < 0.05) (Fig. 3a, Table 2). However, no significant difference in the autoantibody level for HSPD1 was found in GBC (Fig. 3d). As we detected multiple proteins from the single immunoreactive spot, it is possible that HSPD1 was not contributing to immunoreactivity for the specific spot and thus there is a need to explore other proteins identified from the spot. Receiver operating characteristic (ROC) curve analysis of ANXA1 for early stage showed an Area under the ROC Curve (AUC) of 0.69 (95% CI, 0.52–0.86), with 41.7% sensitivity against a specificity of 89.9% in comparison to controls (Fig. 4a), while AUC was 0.5822 for the advanced stage with 15% sensitivity against a specificity of 89.9% in comparison to controls (Table 2).

**Table 2:** Statistical analysis of Dot blot data showing sensitivity or specificity for autoantibody levels for ANXA1

Group comparison	***p*** value	Summary	AUC	Sensitivity (%)	Specificity (%)
GBC stage I and II vs Healthy	0.0177	*	0.6545	25	92.68
GBC stage IIIA vs Healthy	0.0155	*	0.7395	18.18	82.93
GBC stage IIIB vs Healthy	0.0789	ns	0.7561	33.33	87.8
GBC stage IVB vs Healthy	0.2635	ns	0.5483	17.39	92.68
GBC stage I and II vs GSD	0.0007	***	0.724	41.67	95.83
GBC stage IIIA vs GSD	0.0009	***	0.8011	45.45	91.67
GBC stage IIIB vs GSD	0.1043	ns	0.7153	33.33	91.67
GBC stage IVB vs GSD	0.0419	*	0.5951	17.39	91.67
GBC Early stage vs All controls	0.0005	***	0.6919	41.67	89.89
GBC Advance stage vs All controls	0.0206	*	0.5822	15.00	89.89
LN negative vs All controls	< 0.0001	***	0.7306	30.43	89.89
LN positive vs All controls	0.2102	ns	0.5099	13.79	89.89
All cases vs All controls	0.0037	**	0.6075	15.38	95.51

*GBC* Gallbladder carcinomas, *GSD* Gallstone disease, *AUC* area under curve, *CI* confidence interval, *ns* not significant

### IHC analysis

**Fig. 5:**
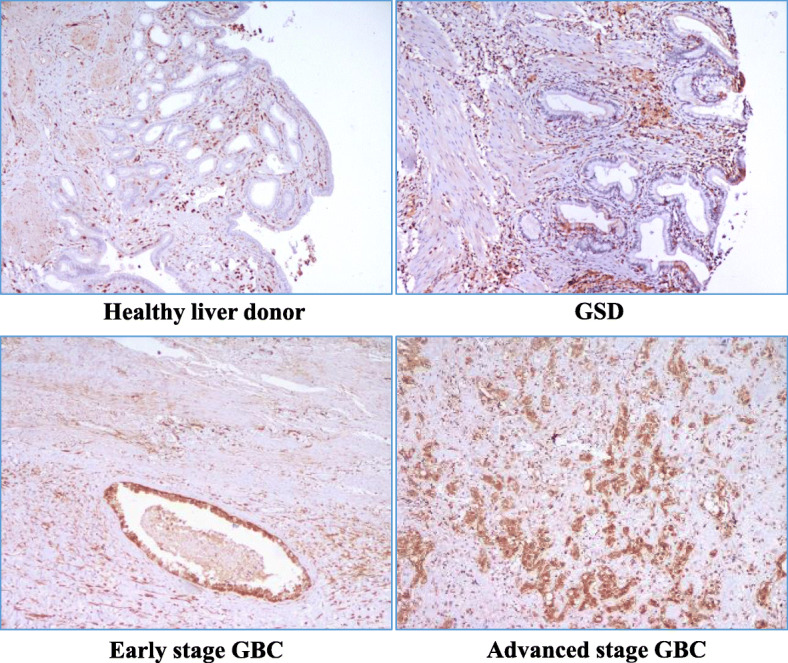
Representative IHC images showing expression of ANXA1 in controls and cases. IHC analysis performed using FFPE tissue from 14 controls (2 Healthy liver donors and 12 GSD cases) and 43 GBC cases (16 early stage and 27 advanced stage GBC cases) showed ‘high’ expression levels in 72% of GBC cases. ANXA1 expression was ‘high’ in 77.7% of advanced stage and 62.5% of early stage GBC cases whereas all the controls showed ‘low’ expression levels. The details of tissue microarrays and individual tissue sections used and IHC procedure is described in the Methods and IHC scoring criteria are shown in Supplementary Table S4

We performed IHC analysis to study the expression of ANXA1 in 14 controls (2 healthy liver donors and 12 GSD cases) and 43 GBC cases (16 early stage and 27 advanced stage GBC cases) and found ‘high’ expression levels in 72% of GBC cases. The expression of ANXA1 was found as ‘high’ in 62.5% of early stage and 77.7% of advance stage cases whereas all the controls showed ‘low’ expression levels (Fig. 5, Supplementary Table S4).

## Discussion

Autoantibody levels in serum against TAAs have shown promise for early detection of cancer [17]. In this study, we have analysed the autoantibody response in GBC. For this purpose, we prepared tumor tissue lysate, as a source of TAAs, from GBC cases (Stage I, II and IIIA) and performed immunodepletion of HSA and IgG to enrich low abundant proteins and reduce non-specific reactivity due to anti-human IgG that was used as a secondary antibody in 1D and 2-D immunoblotting experiments. Immunodepleted tumor tissue proteins were resolved by 2-DE followed by 2-D immunoblot analysis using pooled plasma from controls (healthy volunteers or GSD cases) and GBC cases (stage I and II or IIIA) which led to the identification of 25 protein spots showing high or specific immunoreactivity in GBC. Mass spectrometric analysis (LC-MS/MS) of 8 protein spots that showed intense immunoreactivity in early stage GBC and GBC stage IIIA led to the identification of 27 proteins. A literature survey of the identified proteins was done for their expression and humoral response in GBC or other cancers, based on which we selected proteins for verifications using individual plasma samples.

We focused on proteins identified from four of the intense immunoreactive spots (spot no. 9–12) detected in early stage GBC. Out of 17 proteins identified from these four spots, increased autoantibody levels have been reported against seven of them including ANXA1, HSPD1, CA1, CA2, ALDOA, CTSD, and ECH1. One of these proteins, ANXA1, is previously reported to be overexpressed in GBC tissue in comparison to peri-tumoral tissue (PTs), adenomatous polyp and chronic cholecystitis (CC)**.** The level of ANXA1 was found to be significantly lower in early stage GBC than those in the advanced GBC cases [18]. ANXA1 is also reported to be overexpressed in CCA and could distinguish CCA from HCC [19]. ANXA1 is a calcium- and phospholipid-binding protein involved in cellular signal transduction pathways associated with inflammation, cell differentiation and cell proliferation [20]. Dysregulation and altered localization of ANXA1 have been correlated with tumor development and progression in several cancers including oral squamous cell carcinoma and gastric adenocarcinoma. It is also reported to promote tumor invasion and metastasis [21]. Autoantibody response against ANXA1 is previously reported in lung cancer and is one of the proteins among the panel of six TAAs (p53, NY-ESO1, Annexin I, CAGE, GBU4–5, and SOX2) that can detect up to 40% of all lung cancers in the disease groups, with a specificity of 90% [22, 23]. Linear peptide antigen from ANXA1 analyzed by enzyme-linked immunosorbent assay (ELISA) showed a significant increase in antibody levels in lung and breast cancer [24, 25].

In view of the above, we selected ANXA1 for clinical verifications and analyzed autoantibody levels against recombinant ANXA1 in early and advanced stage GBC cases using Dot blot assay. The results showed a significant increase in autoantibody levels against ANXA1 in early stage GBC (stage I and II) in comparison to controls **(**Fig. 3b, Table 2). Receiver operating characteristic (ROC) curve analysis for ANXA1 showed an Area under the ROC Curve (AUC) of 0.69 (95% CI: 0.52–0.86), with 41.7% sensitivity against a specificity of 89.9% in comparison to controls (Fig. 4a). GBC stage I, II and IIIA are lymph node (LN) negative or non-metastatic stages where tumor cells are not spread to nearby lymph nodes. LN metastasis is the earliest sign of metastatic spread and is one of the established predictors of poor outcome in GBC patients [26]. We also compared the autoantibody levels for ANXA1 in LN negative (GBC stage I, II and IIIA) and LN positive (GBC stage IIIB, IVB) cases and found a significant increase of autoantibody levels in LN negative GBC cases. Receiver operating characteristic (ROC) curve analysis for ANXA1 showed an Area under the ROC Curve (AUC) of 0.73 (95% CI: 0.61–0.85), with 30.4% sensitivity against a specificity of 89.9% in comparison to controls **(**Fig. 4b**)**. The LN positive GBC cases did not show any significant increase in autoantibody levels of ANXA1 (Fig. 3c, Table 2), possibly due to lower immunogenicity of cancer cells in LN metastatic cases [27, 28]. The mechanism for the elevation of IgG antibodies for ANXA1 is not clear. The autoantibody levels and clinical parameters like total leukocyte count (TLC), liver enzymes, cholestasis were compared (Supplementary Figure S1) and we did not find any correlation of the high or low autoantibody levels with any of the clinical parameters (Supplementary Table S5) suggesting that increased autoantibody levels in GBC patients were independent of these parameters and was possibly specific to cancer. There are independent reports on increased expression levels [18, 19] or increased autoantibodies for ANXA1 [22, 23] in several cancers, however, there is no report on a correlation between increased expression levels and autoantibody levels. In the present study, IHC analysis showed ‘high’ expression of ANXA1 in 72% GBC cases while all the controls showed ‘low’ expression levels suggesting that increased expression levels of ANXA1 may have a role in eliciting antibody response in GBC. In order to find if there is any correlation between ANXA1 expression and autoantibody levels, we compared the expression of ANXA1 in 16 GBC cases (8 with high autoantibody levels and 8 with low autoantibody levels against ANXA1). We did not find any correlation of ANXA1 expression and autoantibody levels suggesting that there may be other factors such as post-translational modification or altered localization in tumor cells, for increased autoantibody levels against ANXA1 in GBC.

On similar lines, we also selected another protein HSPD1 for verification. HSPD1 is a chaperonin, involved in regulating apoptosis in cancer. An increased autoantibody level against HSPD1 has been reported in HCC and breast cancer and proposed as a marker for early detection [29–31]. However, our results did not show any significant difference in autoantibody levels in GBC (Fig. 3e and f), thus enhancing the importance of the specific observation made with ANXA1 and the need to investigate other individual proteins detected in the same spot.

The sample size of early stage GBC cases used for the study was small and the analysis could be expanded with a larger cohort of clinical samples for higher confidence in the results. Also, other individual proteins identified from the immunoreactive protein spots such as CA1, CA2, ALDOA may be analyzed for the autoantibody levels in GBC cases to expand the panel of proteins with improved sensitivity to detect GBC at early stage.

## Conclusions

This study involves the application of SERPA approach for the detection of protein spots with high or specific immunoreactivity in early stage GBC. Our data on clinical verification revealed significantly high autoantibody levels against ANXA1 in early stage GBC in comparison to controls suggesting its potential for early detection of GBC. The analysis may be carried out further in a larger cohort of samples for clinical applications.

## Supplementary Information

**Additional file 1: Supplementary Figure S1.** Scatter plot showing variations in TLC, liver enzymes, bilirubin and cholestasis among the cases and controls used for the study. (A) TLC (B) Bilirubin (C) SGOT (D) SGPT (E) ALP levels in samples used for pooling of plasma or tissue for the discovery phase and individual plasma samples for all GSD and GBC cases used for verification study. The dotted line represents the normal levels of these parameters. The solid line represents the bilirubin levels > 2 mg/dL suggests cholestasis. The data was available for ~ 68% of the samples i.e. 33 out of 48 GSD cases and 35 out of 52 GBC cases used for the study and was used for analysis. Normal levels for TLC- 4000–10,000 per mm^3^, Bilirubin- 0.3-1.2 mg/dL, SGOT- > 35 U/L, SGPT- > 35 U/L and ALP- 30-120 U/L. TLC- Total leukocyte count, SGOT-Serum Glutamic Oxaloacetic Transaminase or Aspartate transaminase, SGPT- Serum glutamic pyruvic transaminase or alanine aminotransferase, ALP- Alkaline phosphatase, GSD- Gallstone disease, GBC- Gallbladder cancer.

**Additional file 2: Supplementary Figure S2.** Dot blot images showing autoantibody levels of ANXA1 and HSPD1 in cases and control groups. For Dot blot assay, a total of 75 ng recombinant protein was spotted on PVDF membrane followed by blocking and incubation with individual blood plasma samples from 82 controls (healthy individuals and GSD) and 52 cases (early and advanced stage GBC) overnight at 4 °C. The blots were then incubated with secondary antibody (anti-human IgG HRP conjugated) followed by development using SuperSignal™ West Femto Maximum Sensitivity Substrate (Thermo). The images were acquired using Chemidoc MP (Bio-Rad, USA) and densitometric analysis was performed using ImageLab software version 4.1 (Bio-Rad, USA). The relative levels of autoantibodies in controls and cases are represented as scatter plot in Fig. 3. H: Healthy; GSD: Gallstone disease; I + II: GBC stage I and II; IIIA: GBC stage IIIA; IIIB: GBC stage IIIB; IVB: GBC Stage IVB. The full-length blot images are presented in Supplementary Figure S3.

**Additional file 3: Supplementary Figure S3.** The full-length blot images of Fig. 2 and Fig. S2. (A) The full-length blot images of Fig. 2a. The cropped image includes the lanes (‘Healthy’, ‘GSD’, ‘GBC stage I and II’, ‘GBC stage IIIA’ and ‘Negative’) from full-length blot image with ‘high exposure’. The lane with MW marker is from the blot image with ‘low exposure’. All these blots were developed together. The cropping of the image is indicated with red dashed line (B) The full-length blot images of Fig. 2c. Immunodepleted tumor tissue proteins were resolved by 2-DE and electro transferred onto PVDF membrane. The blots were separately incubated with pooled plasma from healthy individuals or GSD cases or GBC Stage I and II or GBC stage IIIA cases. The blots were first incubated with anti-human IgG conjugated with HRP (i), developed using ECL kit and image was acquired. The same blot was subsequently developed after incubation with StrepTactin-HRP Conjugate (ii) to visualize the MW marker. The full-length 2-D blot image (i) and the lane with MW marker (ii) was cropped and aligned appropriately for inclusion in the Fig. 2c. The cropping of the blot images is indicated with red dashed line. (C) The full-length Dot blot images of Fig. S2. The Dot blot assay for 52 cases and 89 controls was performed in 4 independent sets (Set 1–4). Two recombinant proteins, HSPD1 and ANXA1, were spotted on the same PVDF membrane and exposed to individual plasma samples. The reactive spot areas on each dot blot were quantified as described under Methods and the spots cropped to align the data for HSPD1 and ANXA1 separately as shown in Fig. S2.

**Additional file 4: Supplementary Table S1.** List of identified proteins from immunoreactive protein spots by mass spectrometric analysis.

**Additional file 5: Supplementary Table S2**. List of proteins and peptides identified by mass spectrometric analysis from immunoreactive protein spots.

**Additional file 6: Supplementary Table S3.** Molecular functions of proteins identified by mass spectrometric analysis. Molecular functions are derived from HPRD database [http://www.hprd.org; Ref. [16]] and uniprot database [www.uniprot.org/uniprot].

**Additional file 7: Supplementary Table S4.** Expression of ANXA1 in control and GBC tissue using IHC analysis. IHC was performed on formalin-fixed paraffin-embedded (FFPE) tissue microarrays (TMAs) and individual tissue sections. Two in-house TMA blocks were constructed using the FFPE blocks and included 14 controls (2 healthy liver donors and 12 GSD cases) and 31 GBC cases (9 early stage and 22 advanced stage). Each TMA block consisted of 22 cores of 2 mm diameter and 4 μm sections were cut from the TMA block for carrying out IHC. Individual tissue sections (FFPE) of GBC (7 early stage and 5 advanced stage) were also for IHC analysis. The staining intensity of cancer cells was scored as 0, 1+, 2+/ 3+ indicating negative, low, and strong staining, respectively. All the cases showed 2+/ 3+ staining intensity. The distribution of staining of cancer cells was scored as 0 (less than 10% of cells staining), 1+ (10- < 25% of cell staining), 2+ (25- < 50% of cells staining) and 3+ (≥50% of cells staining). ANXA1 expression was considered ‘high’ if the percentage distribution was ≥25% and ‘low’ if it was < 25%. IHC data analysis was done by two independent pathologists.

**Additional file 8: Supplementary Table S5.** Correlation of the autoantibody levels with clinical parameters TLC, Bilirubin, SGOT, SGPT, ALP levels in GBC cases. We did not find any correlation of increased autoantibody levels with increased levels of TLC, liver enzymes. The values in bold are above the normal range.

## Data Availability

All data generated or analysed during this study are included in this published article and its supplementary information files.

## Abbreviations

GBC: Gallbladder carcinoma
GSD: Gallstone disease
CCA: Cholangiocarcinomas
TAAs: Tumor-associated antigens
LN: Lymph node
SERPA: Serological proteome analysis
2-DE: Two-dimensional gel electrophoresis
LC-MS/MS: Liquid chromatography-tandem mass spectrometry
HCD: Higher-energy collision dissociation
FDR: False discovery rate
ANXA1: Annexin A1
HSPD1: 60 kDa Heat shock protein, mitochondrial
