# Supplementary material for: Immunoproteomics approach revealed elevated autoantibody levels against ANXA1 in early stage gallbladder carcinoma
Source: BMC Cancer. 2020 Dec 1;20:1175. doi: 10.1186/s12885-020-07676-6 (PMC7709428; doi:10.1186/s12885-020-07676-6)
Supplement: Supplementary file 1 — Additional file 1: Supplementary Figure S1. Scatter plot showing variations in TLC, liver enzymes, bilirubin and cholestasis among the cases and controls used for the study. (A) TLC (B) Bilirubin (C) SGOT (D) SGPT (E) ALP levels in samples used for pooling of plasma or tissue for the discovery phase and individual plasma samples for all GSD and GBC cases used for verification study. The dotted line represents the normal levels of these parameters. The solid line represents the bilirubin levels > 2 mg/dL suggests cholestasis. The data was available for ~ 68% of the samples i.e. 33 out of 48 GSD cases and 35 out of 52 GBC cases used for the study and was used for analysis. Normal levels for TLC- 4000–10,000 per mm3, Bilirubin- 0.3-1.2 mg/dL, SGOT- > 35 U/L, SGPT- > 35 U/L and ALP- 30-120 U/L. TLC- Total leukocyte count, SGOT-Serum Glutamic Oxaloacetic Transaminase or Aspartate transaminase, SGPT- Serum glutamic pyruvic transaminase or alanine aminotransferase, ALP- Alkaline phosphatase, GSD- Gallstone disease, GBC- Gallbladder cancer. [file 12885_2020_7676_MOESM1_ESM.pptx]

## Slide 1
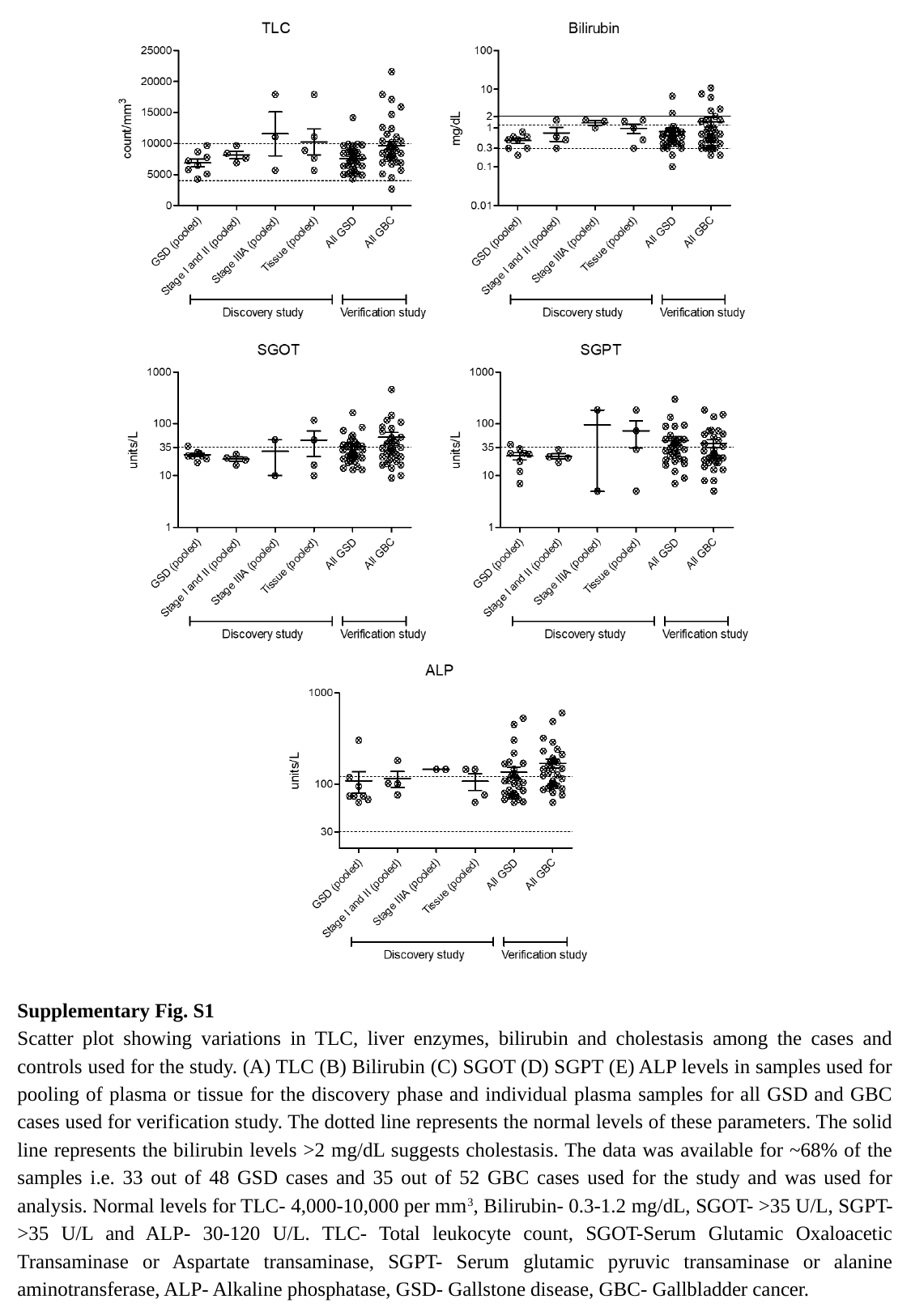

Supplementary Fig. S1
Scatter plot showing variations in TLC, liver enzymes, bilirubin and cholestasis among the cases and controls used for the study. (A) TLC (B) Bilirubin (C) SGOT (D) SGPT (E) ALP levels in samples used for pooling of plasma or tissue for the discovery phase and individual plasma samples for all GSD and GBC cases used for verification study. The dotted line represents the normal levels of these parameters. The solid line represents the bilirubin levels >2 mg/dL suggests cholestasis. The data was available for ~68% of the samples i.e. 33 out of 48 GSD cases and 35 out of 52 GBC cases used for the study and was used for analysis. Normal levels for TLC- 4,000-10,000 per mm3, Bilirubin- 0.3-1.2 mg/dL, SGOT- >35 U/L, SGPT- >35 U/L and ALP- 30-120 U/L. TLC- Total leukocyte count, SGOT-Serum Glutamic Oxaloacetic Transaminase or Aspartate transaminase, SGPT- Serum glutamic pyruvic transaminase or alanine aminotransferase, ALP- Alkaline phosphatase, GSD- Gallstone disease, GBC- Gallbladder cancer.
