# Supplementary material for: Immunoproteomics approach revealed elevated autoantibody levels against ANXA1 in early stage gallbladder carcinoma
Source: BMC Cancer. 2020 Dec 1;20:1175. doi: 10.1186/s12885-020-07676-6 (PMC7709428; doi:10.1186/s12885-020-07676-6)
Supplement: Supplementary file 2 — Additional file 2: Supplementary Figure S2. Dot blot images showing autoantibody levels of ANXA1 and HSPD1 in cases and control groups. For Dot blot assay, a total of 75 ng recombinant protein was spotted on PVDF membrane followed by blocking and incubation with individual blood plasma samples from 82 controls (healthy individuals and GSD) and 52 cases (early and advanced stage GBC) overnight at 4 °C. The blots were then incubated with secondary antibody (anti-human IgG HRP conjugated) followed by development using SuperSignal™ West Femto Maximum Sensitivity Substrate (Thermo). The images were acquired using Chemidoc MP (Bio-Rad, USA) and densitometric analysis was performed using ImageLab software version 4.1 (Bio-Rad, USA). The relative levels of autoantibodies in controls and cases are represented as scatter plot in Fig. 3. H: Healthy; GSD: Gallstone disease; I + II: GBC stage I and II; IIIA: GBC stage IIIA; IIIB: GBC stage IIIB; IVB: GBC Stage IVB. The full-length blot images are presented in Supplementary Figure S3. [file 12885_2020_7676_MOESM2_ESM.pptx]

## Slide 1
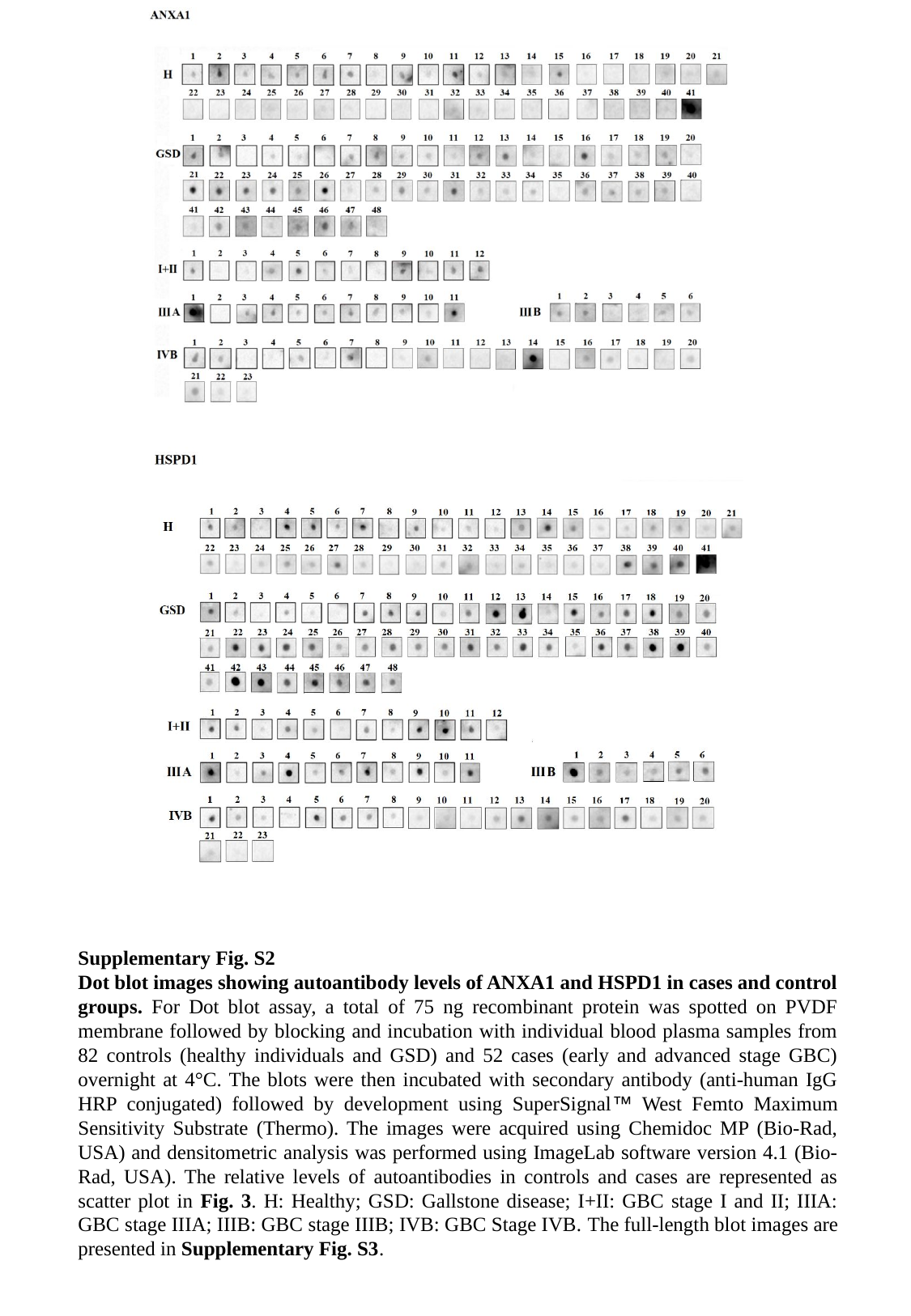

Supplementary Fig. S2
Dot blot images showing autoantibody levels of ANXA1 and HSPD1 in cases and control groups. For Dot blot assay, a total of 75 ng recombinant protein was spotted on PVDF membrane followed by blocking and incubation with individual blood plasma samples from 82 controls (healthy individuals and GSD) and 52 cases (early and advanced stage GBC) overnight at 4°C. The blots were then incubated with secondary antibody (anti-human IgG HRP conjugated) followed by development using SuperSignal™ West Femto Maximum Sensitivity Substrate (Thermo). The images were acquired using Chemidoc MP (Bio-Rad, USA) and densitometric analysis was performed using ImageLab software version 4.1 (Bio-Rad, USA). The relative levels of autoantibodies in controls and cases are represented as scatter plot in Fig. 3. H: Healthy; GSD: Gallstone disease; I+II: GBC stage I and II; IIIA: GBC stage IIIA; IIIB: GBC stage IIIB; IVB: GBC Stage IVB. The full-length blot images are presented in Supplementary Fig. S3.
