# Supplementary material for: Immunoproteomics approach revealed elevated autoantibody levels against ANXA1 in early stage gallbladder carcinoma
Source: BMC Cancer. 2020 Dec 1;20:1175. doi: 10.1186/s12885-020-07676-6 (PMC7709428; doi:10.1186/s12885-020-07676-6)
Supplement: Supplementary file 3 — Additional file 3: Supplementary Figure S3. The full-length blot images of Fig. 2 and Fig. S2. (A) The full-length blot images of Fig. 2a. The cropped image includes the lanes (‘Healthy’, ‘GSD’, ‘GBC stage I and II’, ‘GBC stage IIIA’ and ‘Negative’) from full-length blot image with ‘high exposure’. The lane with MW marker is from the blot image with ‘low exposure’. All these blots were developed together. The cropping of the image is indicated with red dashed line (B) The full-length blot images of Fig. 2c. Immunodepleted tumor tissue proteins were resolved by 2-DE and electro transferred onto PVDF membrane. The blots were separately incubated with pooled plasma from healthy individuals or GSD cases or GBC Stage I and II or GBC stage IIIA cases. The blots were first incubated with anti-human IgG conjugated with HRP (i), developed using ECL kit and image was acquired. The same blot was subsequently developed after incubation with StrepTactin-HRP Conjugate (ii) to visualize the MW marker. The full-length 2-D blot image (i) and the lane with MW marker (ii) was cropped and aligned appropriately for inclusion in the Fig. 2c. The cropping of the blot images is indicated with red dashed line. (C) The full-length Dot blot images of Fig. S2. The Dot blot assay for 52 cases and 89 controls was performed in 4 independent sets (Set 1–4). Two recombinant proteins, HSPD1 and ANXA1, were spotted on the same PVDF membrane and exposed to individual plasma samples. The reactive spot areas on each dot blot were quantified as described under Methods and the spots cropped to align the data for HSPD1 and ANXA1 separately as shown in Fig. S2. [file 12885_2020_7676_MOESM3_ESM.pptx]

## Slide 1
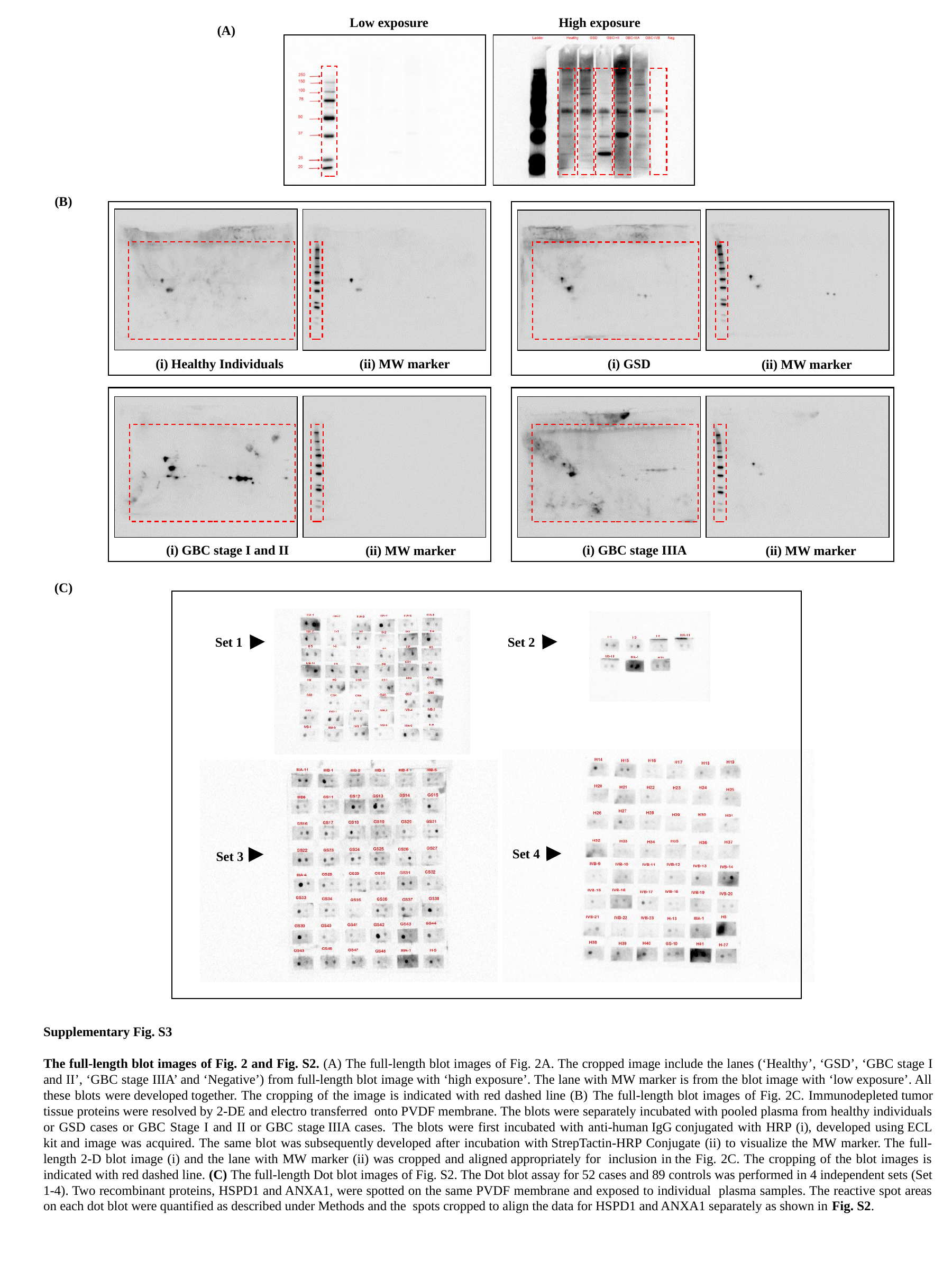

Low exposure
High exposure
(A)
(B)
(i) Healthy Individuals
(ii) MW marker
(i) GSD
(ii) MW marker
(i) GBC stage I and II
(i) GBC stage IIIA
(ii) MW marker
(ii) MW marker
(C)
Set 1
Set 2
Set 4
Set 3
Supplementary Fig. S3
The full-length blot images of Fig. 2 and Fig. S2. (A) The full-length blot images of Fig. 2A. The cropped image include the lanes (‘Healthy’, ‘GSD’, ‘GBC stage I and II’, ‘GBC stage IIIA’ and ‘Negative’) from full-length blot image with ‘high exposure’. The lane with MW marker is from the blot image with ‘low exposure’. All these blots were developed together. The cropping of the image is indicated with red dashed line (B) The full-length blot images of Fig. 2C. Immunodepleted tumor tissue proteins were resolved by 2-DE and electro transferred  onto PVDF membrane. The blots were separately incubated with pooled plasma from healthy individuals or GSD cases or GBC Stage I and II or GBC stage IIIA cases.  The blots were first incubated with anti-human IgG conjugated with HRP (i), developed using ECL kit and image was acquired. The same blot was subsequently developed after incubation with StrepTactin-HRP Conjugate (ii) to visualize the MW marker. The full-length 2-D blot image (i) and the lane with MW marker (ii) was cropped and aligned appropriately for  inclusion in the Fig. 2C. The cropping of the blot images is indicated with red dashed line. (C) The full-length Dot blot images of Fig. S2. The Dot blot assay for 52 cases and 89 controls was performed in 4 independent sets (Set 1-4). Two recombinant proteins, HSPD1 and ANXA1, were spotted on the same PVDF membrane and exposed to individual plasma samples. The reactive spot areas on each dot blot were quantified as described under Methods and the spots cropped to align the data for HSPD1 and ANXA1 separately as shown in Fig. S2.
