# Supplementary material for: Immunoproteomics approach revealed elevated autoantibody levels against ANXA1 in early stage gallbladder carcinoma
Source: BMC Cancer. 2020 Dec 1;20:1175. doi: 10.1186/s12885-020-07676-6 (PMC7709428; doi:10.1186/s12885-020-07676-6)
Supplement: Supplementary file 4 — Additional file 4: Supplementary Table S1. List of identified proteins from immunoreactive protein spots by mass spectrometric analysis. [file 12885_2020_7676_MOESM4_ESM.docx]

**Supplementary Table S1**

List of identified proteins from immunoreactive protein spots by mass spectrometric analysis.

| **Spot No.** | **Mol. weight**  **/ PI** | **Gene Symbols of proteins** |
| --- | --- | --- |
| 9 | 31.4/ 7.25 | ALDOB |
| 10 | 30.8/ 7.7 | CA1, CTSD, ALDOB, ALDOA, ECH1, IGLL5, HBB, PSMA4, PLEC |
| 11 | 29.8/ 8.05 | CA2, CA1, HBB, HAGH, PGAM1, IGLL5, ANXA1, CTSD, FLNB, PLEC |
| 12 | 29.2/ 8.3 | HBB, CA2, CA1, IGLL5, ECH1, ALDOB, HSPD1, AK2, HBA1, ANXA1, HAGH, PLEC |
| 17 | 41.7/ 8.25 | ALDOB, ALDOA, OTC, CAT, LTF |
| 18 | 41.7/ 8.5 | ALDOB, ARG1, CAT, LTF |
| 19 | 41.9/ 8.7 | ALDOB, ALDOA |
| 20 | 42.2/ 8.95 | ALDOB, ALDOA, PGK1, HAO1, GPI, ACAT1, ADH1B, MDH2, LTF |
