# Supplementary material for: Immunoproteomics approach revealed elevated autoantibody levels against ANXA1 in early stage gallbladder carcinoma
Source: BMC Cancer. 2020 Dec 1;20:1175. doi: 10.1186/s12885-020-07676-6 (PMC7709428; doi:10.1186/s12885-020-07676-6)
Supplement: Supplementary file 6 — Additional file 6: Supplementary Table S3. Molecular functions of proteins identified by mass spectrometric analysis. Molecular functions are derived from HPRD database [http://www.hprd.org; Ref. [16]] and uniprot database [www.uniprot.org/uniprot]. [file 12885_2020_7676_MOESM6_ESM.docx]

**Supplementary Table S3**

**Molecular functions of proteins identified by mass spectrometric analysis.** Molecular functions are derived from HPRD database [http://www.hprd.org; Ref. 16] and uniprot database [www.uniprot.org/uniprot].

| **Gene Symbol** | **Protein Name** | **Unique Peptides** | **PSM** | **Primary Localization** | **Alternate Localization** | **Molecular Function** | **Biological Process** |
| --- | --- | --- | --- | --- | --- | --- | --- |
| ACAT1 | Acetyl-coa acetyltransferase, mitochondrial precursor | 3 | 6 | Mitochondrion | Cytoplasm | Acyltransferase activity | Metabolism;  Energy pathways |
| ADH1B | Alcohol dehydrogenase 1B isoform 2 | 2 | 9 | Mitochondrion | - | Catalytic activity | Metabolism;  Energy pathways |
| AK2 | Adenylate kinase 2, mitochondrial isoform h | 2 | 4 | Mitochondrion | Cytoplasm | Catalytic activity | Metabolism;  Energy pathways |
| ALDOA | Fructose-bisphosphate aldolase A isoform 1 | 3 | 6 | Cytoplasm | Cytoplasm,  Mitochondrion, Nucleus | Lyase activity | Metabolism;  Energy pathways |
| ALDOB | Fructose-bisphosphate aldolase B | 2 | 2 | Cytoplasm | - | Lyase activity | Metabolism;  Energy pathways |
| ANXA1 | Annexin A1 | 2 | 7 | Plasma membrane | Cytoplasm,  Mitochondrion, Nucleus | Calcium ion binding | Cell communication;  Signal transduction |
| ARG1 | Arginase-1 isoform 2 | 4 | 6 | Cytoplasm, Plasma membrane | [Extracellular](http://www.ncbi.nlm.nih.gov/entrez/query.fcgi?cmd=Retrieve&db=PubMed&list_uids=16188874&dopt=Abstract) | Hydrolase activity | Metabolism;  Energy pathways |
| CA1 | Carbonic anhydrase 1 isoform a | 8 | 81 | Cytoplasm | - | Catalytic activity | Metabolism;  Energy pathways |
| CA2 | Carbonic anhydrase 2 isoform 1 | 9 | 68 | Cytoplasm | Plasma membrane, Golgi apparatus, Nucleus, Extracellular | Catalytic activity | Metabolism;  Energy pathways |
| CAT | Catalase | 2 | 5 | Cytoplasm | Mitochondrion, Peroxisome | Oxidoreductase activity | Metabolism;  Energy pathways |
| CTSD | Cathepsin D preproprotein | 5 | 19 | Lysosome | Extracellular, Golgi apparatus, Endoplasmic reticulum, Mitochondrion | Aspartic-type signal peptidase activity | Protein metabolism |
| ECH1 | Delta(3,5)-Delta(2,4)-dienoyl-coa isomerase, mitochondrial precursor | 3 | 6 | Peroxisome | Mitochondrion, Cytoplasm | Catalytic activity | Metabolism;  Energy pathways |
| FLNB | Filamin-B isoform 4 | 2 | 3 | Cytoplasm | Nucleus, Plasma membrane | Cytoskeletal protein binding | Cell growth and/or maintenance |
| GPI | Glucose-6-phosphate isomerase isoform 4 | 4 | 9 | Cytoplasm | Mitochondrion | Isomerase activity | Metabolism;  Energy pathways |
| HAGH | Hydroxyacylglutathione hydrolase, mitochondrial isoform 2 | 4 | 8 | Cytoplasm | Mitochondrion | Hydrolase activity | Metabolism;  Energy pathways |
| HAO1 | Hydroxyacid oxidase 1 | 5 | 11 | Peroxisome | - | Catalytic activity | Metabolism;  Energy pathways |
| HBA1 | Hemoglobin subunit alpha | 2 | 5 | Cytosol | Extracellular | Transporter activity | Transport |
| HBB | Hemoglobin subunit beta | 2 | 5 | Extracellular | - | Transporter activity | Transport |
| HSPD1 | 60 kda heat shock protein, mitochondrial | 3 | 7 | Mitochondrial matrix | Mitochondrion, Cytoplasm, Cytosol, Plasma membrane, Endoplasmic reticulum, Peroxisome, Secretory granule, Extracellular, Golgi apparatus, Nucleus | Heat shock protein activity | Protein folding; Apoptosis; Regulation of immune response; Signal transduction |
| IGLL5 | Immunoglobulin lambda-like polypeptide 5 isoform 1 | 2 | 6 | Extracellular | - | antigen binding | B cell receptor signaling pathway |
| LTF | Lactotransferrin isoform 2 | 2 | 3 | [Secretory granule](http://www.ncbi.nlm.nih.gov/entrez/query.fcgi?cmd=Retrieve&db=PubMed&list_uids=3028636&dopt=Abstract) | Golgi apparatus, Endoplasmic reticulum, Nucleolus,  Plasma membrane, Cytoplasm,  Extracellular, Nucleus | Transporter activity | Transport |
| MDH2 | Malate dehydrogenase, mitochondrial isoform 3 | 2 | 3 | Mitochondrion | Nucleus, Extracellular | [Catalytic activity](http://godatabase.org/cgi-bin/go.cgi?query=GO:0003824&view=details&search_constraint=terms&depth=0) | Metabolism;  Energy pathways |
| OTC | Ornithine carbamoyltransferase, mitochondrial precursor | 2 | 3 | Mitochondrion | Cytoplasm | Carboxyl- and carbamoyl transferase activity | Metabolism;  Energy pathways |
| PGAM1 | Phosphoglycerate mutase 1 isoform 2 | 3 | 8 | Cytoplasm | - | Catalytic activity | Metabolism;  Energy pathways |
| PGK1 | Phosphoglycerate kinase 1 | 10 | 38 | Cytoplasm | [Cytoplasmic vesicle,  Nucleus, Mitochondrion](http://www.ncbi.nlm.nih.gov/entrez/query.fcgi?cmd=Retrieve&db=PubMed&list_uids=12488440&dopt=Abstract) | Catalytic activity | Metabolism;  Energy pathways |
| PLEC | Plectin isoform 1d | 2 | 2 | Cytoskeleton | Hemidesmosome | Actin binding | Hemidesmosome assembly |
| PSMA4 | Proteasome subunit alpha type-4 isoform 2 | 2 | 2 | Cytoplasm | Endoplasmic reticulum,  Nucleus | Ubiquitin-specific protease activity | Protein metabolism |
