# Supplementary material for: Immunoproteomics approach revealed elevated autoantibody levels against ANXA1 in early stage gallbladder carcinoma
Source: BMC Cancer. 2020 Dec 1;20:1175. doi: 10.1186/s12885-020-07676-6 (PMC7709428; doi:10.1186/s12885-020-07676-6)
Supplement: Supplementary file 7 — Additional file 7: Supplementary Table S4. Expression of ANXA1 in control and GBC tissue using IHC analysis. IHC was performed on formalin-fixed paraffin-embedded (FFPE) tissue microarrays (TMAs) and individual tissue sections. Two in-house TMA blocks were constructed using the FFPE blocks and included 14 controls (2 healthy liver donors and 12 GSD cases) and 31 GBC cases (9 early stage and 22 advanced stage). Each TMA block consisted of 22 cores of 2 mm diameter and 4 μm sections were cut from the TMA block for carrying out IHC. Individual tissue sections (FFPE) of GBC (7 early stage and 5 advanced stage) were also for IHC analysis. The staining intensity of cancer cells was scored as 0, 1+, 2+/ 3+ indicating negative, low, and strong staining, respectively. All the cases showed 2+/ 3+ staining intensity. The distribution of staining of cancer cells was scored as 0 (less than 10% of cells staining), 1+ (10- < 25% of cell staining), 2+ (25- < 50% of cells staining) and 3+ (≥50% of cells staining). ANXA1 expression was considered ‘high’ if the percentage distribution was ≥25% and ‘low’ if it was < 25%. IHC data analysis was done by two independent pathologists. [file 12885_2020_7676_MOESM7_ESM.docx]

**Supplementary Table S4**

**Expression of ANXA1 in control and GBC tissue using IHC analysis.** IHC was performed on formalin-fixed paraffin-embedded (FFPE) tissue microarrays (TMAs) and individual tissue sections. Two in-house TMA blocks were constructed using the FFPE blocks and included 14 controls (2 healthy liver donors and 12 GSD cases) and 31 GBC cases (9 early stage and 22 advanced stage). Each TMA block consisted of 22 cores of 2 mm diameter and 4 µm sections were cut from the TMA block for carrying out IHC. Individual tissue sections (FFPE) of GBC (7 early stage and 5 advanced stage) were also for IHC analysis. The staining intensity of cancer cells were scored as 0, 1+, 2+/ 3+ indicating negative, low, and strong staining, respectively. All the cases showed 2+/ 3+ staining intensity. The distribution of staining of cancer cells was scored as 0 (less than 10% of cells staining), 1+ (10-<25% of cell staining), 2+ (25-<50% of cells staining) and 3+ (≥50% of cells staining). ANXA1 expression was considered ‘high’ if percentage distribution was ≥25% and ‘low’ if it was <25%. IHC data analysis was done by two independent pathologists.

| Group | Total samples | ANXA1 expression (No. of cases/ %) | |
| --- | --- | --- | --- |
|  |  | ‘Low’ | ‘High’ |
| Healthy liver donor | 2 | 2/ 100% | Nil |
| GSD | 12 | 12/ 100% | Nil |
| Early stage GBC | 16 | 6/ 37.5% | 10/ 62.5% |
| Advanced stage GBC | 27 | 6/ 23% | 21/ 77.7% |
| All GBC Cases | 43 | 12/ 27.9% | 31/ 72% |
