# Supplementary material for: Immunoproteomics approach revealed elevated autoantibody levels against ANXA1 in early stage gallbladder carcinoma
Source: BMC Cancer. 2020 Dec 1;20:1175. doi: 10.1186/s12885-020-07676-6 (PMC7709428; doi:10.1186/s12885-020-07676-6)
Supplement: Supplementary file 8 — Additional file 8: Supplementary Table S5. Correlation of the autoantibody levels with clinical parameters TLC, Bilirubin, SGOT, SGPT, ALP levels in GBC cases. We did not find any correlation of increased autoantibody levels with increased levels of TLC, liver enzymes. The values in bold are above the normal range. [file 12885_2020_7676_MOESM8_ESM.docx]

**Supplementary Table S5**

Correlation of the autoantibody levels with clinical parameters TLC, Bilirubin, SGOT, SGPT, ALP levels in GBC cases. We did not find any correlation of increased autoantibody levels with increased levels of TLC, liver enzymes. The values in bold are above the normal range.

| **S. No.** | **Anti ANXA1 Autoantibody level** | **Anti ANXA1 Autoantibody level (*percentile value)** | **TLC** | **Bilirubin** | **SGOT** | **SGPT** | **ALP** | **Cholestasis** |
| --- | --- | --- | --- | --- | --- | --- | --- | --- |
|  | High Aab | 20.786 | **17900** | **1.6** | - | - | - | No |
|  | High Aab | 22.716 | **15900** | 0.2 | 14 | 8 | **124** | No |
|  | High Aab | 21.359 | 8400 | **1.6** | 20 | 22 | **181** | No |
|  | High Aab | 28.31 | 8000 | 0.7 | 34 | 25 | 96 | No |
|  | Low Aab | 0 | 9800 | 0.8 | 28 | 20 | 87 | No |
|  | Low Aab | 2.872 | 9700 | 0.6 | 26 | 18 | 102 | No |
|  | Low Aab | 2.773 | 8300 | 0.7 | 30 | 27 | **132** | No |
|  | Low Aab | 2.914 | 7650 | 0.5 | 16 | 32 | 76 | No |
|  | Low Aab | 2.154 | 7000 | 0.8 | **41** | **37** | 89 | No |

*The autoantibody levels are indicated by density (arbitrary units) of immunoreactive spots (percentile value) after dot blot assay. The percentile value below 20 was considered ‘low’ and >20 was considered ‘high’. The patients include 8 females and 1 male with age range of 34 to 66 years.

TLC- Total leukocyte count, SGOT-Serum Glutamic Oxaloacetic Transaminase or Aspartate transaminase, SGPT- Serum glutamic pyruvic transaminase or alanine aminotransferase, ALP- Alkaline phosphatase, GSD- Gallstone disease, GBC- Gallbladder cancer
